# Feasibility and Acceptability of a Mobile App–Based TEAM-CBT (Testing Empathy Assessment Methods–Cognitive Behavioral Therapy) Intervention (Feeling Good) for Depression: Secondary Data Analysis

**DOI:** 10.2196/52369

**Published:** 2024-05-10

**Authors:** Nicholas Bisconti, Mackenzie Odier, Matthew Becker, Kim Bullock

**Affiliations:** 1 PGSP-Stanford PsyD. Consortium Palo Alto, CA United States; 2 Stanford School of Medicine Department of Psychiatry and Behavioral Sciences Stanford University Palo Alto, CA United States

**Keywords:** depression, mobile health, mHealth, cognitive behavioral therapy, mobile phone

## Abstract

**Background:**

The Feeling Good App is an automated stand-alone digital mobile mental health tool currently undergoing beta testing with the goal of providing evidence-informed self-help lessons and exercises to help individuals reduce depressive symptoms without guidance from a mental health provider. Users work through intensive basic training (IBT) and ongoing training models that provide education regarding cognitive behavioral therapy principles from a smartphone.

**Objective:**

The key objective of this study was to perform a nonsponsored third-party academic assessment of an industry-generated data set; this data set focused on the safety, feasibility, and accessibility of a commercial automated digital mobile mental health app that was developed to reduce feelings associated with depression.

**Methods:**

The Feeling Good App development team created a waitlist cohort crossover design and measured symptoms of depression and anxiety using the Patient Health Questionnaire-9, Generalized Anxiety Disorder-7, and an app-specific measure of negative feelings called the 7 Dimension Emotion Slider (7-DES). The waitlist cohort crossover design divided the participants into 2 groups, where 48.6% (141/290) of the participants were given immediate access to the apps, while 51.4% (149/290) were placed on a 2-week waitlist before being given access to the app. Data collected by the Feeling Good App development team were deidentified and provided to the authors of this paper for analysis through a nonsponsored university data use agreement. All quantitative data were analyzed using SPSS Statistics (version 28.0; IBM Corp). Descriptive statistics were calculated for demographic variables. Feasibility and acceptability were descriptively assessed. All participants included in the quantitative data were given access to the Feeling Good App; this study did not include a control group.

**Results:**

In terms of safety, there was no statistically significant change in suicidality from preintervention to postintervention time points (*t*_288_=0.0; *P*>.99), and there was a statistically significant decrease in hopelessness from preintervention to postintervention time points (*F*_289_*=*30.16; *P<*.01). In terms of acceptability, 72.2% (166/230) of the users who started the initial 2-day IBT went on to complete it, while 34.8% (80/230) of the users who started IBT completed the entirety of the apps’ 4-week protocol (150/230, 65.22% dropout rate over 4 weeks).

**Conclusions:**

This study is the first reported proof-of-concept evaluation of the Feeling Good App in terms of safety, feasibility, and statistical trends within the data set. It demonstrates a feasible and novel approach to industry and academic collaboration in the process of developing a digital mental health technology translated from an existing evidence-informed treatment. The results support the prototype app as safe for a select nonclinical population. The app had acceptable levels of engagement and dropouts throughout the intervention. Those who stay engaged showed reductions in symptom severity of depression warranting further investigation of the app’s efficacy.

## Introduction

### Background

Depression is a highly prevalent illness that affects approximately 280 million people worldwide [1]. Depression leads to varying degrees of functional impairment and distress [2]. Depression is most often treated through pharmacological intervention, psychotherapy, or neuromodulation with varying efficacy [3]. Suicide is the second leading cause of death in the United States among individuals aged between 10 and 14 years and 25 and 34 years, the third leading cause of death among individuals aged between 15 and 24 years, and the fourth leading cause of death among individuals aged between 35 and 44 years [4,5]. There are approximately 2 times as many suicides (45,979) in the United States as there are homicides (n=24,576) [4]. In pre–COVID-19 times, only 56.8% of people diagnosed with major depressive disorder obtained care. For those who reach out for help, it is estimated that only 37.5% receive minimally adequate treatment or evidence-based psychotherapies [4]. Untreated mental health issues have immense negative effects, from the psychological and physical impacts on the individual to increased health care use costs and loss of productivity that impact communities, societies, and economies [5,6]. Depression is the world’s leading cause of disability, and despite various known and effective treatments, >75% of people in low- and middle-income countries receive no treatment [7]. Many individuals with depression are unable to receive effective treatment for a variety of reasons including due to a lack of resources [2], barriers to access such as systems without enough trained providers, and concerns about privacy and stigma. Providing accessible scalable services at low cost is imperative in combating the global mental health crisis.

Mobile smartphone apps and internet-based treatments present a tremendous opportunity to increase the accessibility of mental health care due to their ability to scale and reduce other barriers to entry [8,9]. A meta-analysis from Serrano-Ripoll et al [10] examined existing app-based interventions for reducing depressive symptoms and found that most of these apps significantly reduced symptoms, with moderate effect sizes (0.51) and significantly larger efficacy in more severe cases. There are a variety of publicly available mobile apps for depression that demonstrate clinically significant decreases in depression. Many interventions use a mixed model of automated multimedia and guided interventions to treat symptoms of depression. These interventions, which use both user-guided material as well as clinician support (nurse-moderated and therapist-guided), are shown to be effective, yet they are limited in their practicality and scale, as they rely heavily on clinician support [11-15]. There is also substantial literature into the efficacy of self-guided apps to treat symptoms of depression and anxiety, with many of the publicly available apps based in basic psychoeducation, mindfulness, and cognitive behavioral therapy (CBT). CBT and mindfulness-based apps, interventions that typically range from 4 to 12 weeks, show significant reductions in symptoms and symptom severity from baseline [16-23].

With >10,000 mental health apps in the market, the choices are overwhelming, and knowing how to evaluate and choose a high-quality app is important. One potential danger in the development and innovations surrounding mental health mobile app–based solutions for depression is that they can be distributed without scientific evidence supporting their treatment techniques [10]. A recent study by Larsen et al [24] showed that in the description of the top 73 mental health apps, 44% used general scientific language such as *evidence-based treatment* while only 2 apps cited low-quality primary evidence and 1 app included a citation to published literature [24]. Differentiating between an app being based in evidence (ie, informed and including principles of evidence-based treatments) and being evidence based (ie, research demonstrating the app is efficacious) is crucial for both app developers and consumers alike [25]. Often, obtaining high-quality evidence is challenging given the cost of industry and academia collaboration in the form of sponsored projects. These projects often occur after the app has been developed and do not leave room for academic or clinical input into industry design during the translational process.

A challenge for mobile mental health solutions is whether an effective app is actually being used. In assessing the usability of a mobile mental health app, one of the primary criteria is adherence. Baumel et al [8] showed that in a study of 100,000 downloads of mental health apps, the median retention rate after 15 days was only 3.9%. In a review of randomized controlled trials of smartphones apps targeting depressive symptoms, Baumel et al [8] found a mean dropout rate of 26.2% that increased to 47.8% when accounting for publication bias. While digital tools with no human interaction have shown to be similarly effective compared with traditional face-to-face treatment, dropout rates are more than twice as high (33% dropout with no interaction) when compared with 11% dropout rates in apps with human interaction [8].

As many conventional evidence-based therapies make the leap to incorporating technology and mobile app use into their dissemination processes, evaluating the efficacy and use of these innovations by independent clinician researchers is crucial. Our group set out to evaluate such a mobile app based on a very popular and common therapy from Dr David Burns’ *Feeling Great,* a self-help CBT book for depression by the Feeling Good Corporation. The Feeling Good App being developed is a smartphone app based upon the principles of Testing Empathy Assessment Methods CBT (TEAM-CBT) [26-29]. TEAM-CBT differs from traditional CBT in four key ways: (1) T=testing, frequent ecological momentary assessment of how users feel in the here-and-now (as opposed to the past 2 weeks) is used to track progress as well as relapses between classes; (2) E=empathy, the use of a variety of validation techniques to make the user feel cared about and understood; (3) A=address resistance, the identification and reduction of motivational process and outcome resistance that nearly always interferes with recovery from depression and anxiety; and (4) M=methods, the use of dozens of techniques that draw from multiple schools of therapy. The techniques are chosen based on the type and categories of problem the user is struggling with. Although TEAM-CBT therapy itself has never been clinically tested, it is considered an evidence-informed therapy incorporating a myriad of evidence-based techniques into its practices.

The current Feeling Good App prototype does not require human intervention and is entirely automated. The current digital TEAM-CBT–based intervention consists of (1) intensive basic training (IBT), which spans 1 to 2 days and interactively teaches principles of cognitive therapy, and (2) ongoing training modules (OTMs) designed to integrate prior lessons and challenge automatic thoughts and core beliefs over 3 to 4 weeks. These training modules include a variety of TEAM-CBT techniques and interactive quizzes to reinforce learning.

### Objective

To begin the process of rigorously testing the efficacy, usability, and scalability of this digital mental health intervention, we engaged in a third-party, nonsponsored proof-of-concept analysis of this company’s data set. The electronic data were reportedly generated from an internally blinded randomized waitlist crossover trial. This trial’s goal was to inform both the app development and help determine whether the technology warranted more resource-intensive sponsored research by academic clinical scientists. This form of research may serve as a model for initial low-risk engagement by academia and industry with minimal conflicts of interest.

The data set was collected during several days of individual digitally delivered intensive basic TEAM-CBT training followed by 4 weeks of additional content to help users challenge distorted thoughts and core beliefs. Using a randomized waitlist crossover data set of a beta test run by and provided to us by the Feeling Good App development team, we examined the safety, feasibility, and acceptability of this digital intervention. In addition, we performed exploratory analyses, examining the evidence for efficacy of this digital app for improving mood symptoms.

## Methods

### Recruitment

Data were collected by the Feeling Good App Corporation from users who were recruited from a waitlist of several thousand individuals interested in participating in a beta test of a TEAM-CBT mobile app mentioned in one of the Feeling Good podcast episodes hosted by David Burns. A deidentified data set was provided to the Department of Psychiatry and Behavioral Sciences, Standford School of Medicine, via a nonsponsored data use agreement with Feeling Good App Corporation. The Feeling Good App development team retained ownership of the original data and allowed permission through the data use agreement for the authors to analyze and publish the findings independently. All data for this study were collected by the Feeling Good App development team and not by the authors of this study. During data collection, authors consulted with the Feeling Good App development team and shared insight on best practices for creating a waitlist crossover cohort design and types of data to collect from users (eg, demographic information, exclusionary criteria, and measures); however, the authors did not have any direct oversight over its collection or means to assess accuracy.

The Feeling Good App was still in development and not commercially available during the study period. All participants were recruited from a waitlist of individuals who had heard of the app from Burns’ social media sites and podcast and requested access. An email invitation was sent to waitlist members, and to those who responded to the email invitation, a consent for use of deidentified data in research and a screening form were emailed. Screening information was collected using a Google Survey. Screened respondents were included if they were aged >18 years, were fluent in English, had regular access to an iPhone, and had no previous experience with the Feeling Good App. They also indicated a willingness to spend at least 4 hours per day on IBT and 30 minutes per day on OTMs. Participants were excluded if they admitted to ever having a suicide attempt, suicidal urges, or plans, or self-harm behavior engagement or urges to harm others. Participants who indicated “yes” to any of these risk and safety questions were excluded from the study at enrollment and encouraged to seek help from a mental health professional and given resources to do so.

### Ethical Considerations

Use and analysis of the data set was approved by the Stanford institutional review board (protocol ID 67718; Palo Alto, CA).

### Intervention

After recruitment and screening, participants obtained access to the contents of the app by invitation to download the iOS app onto their Apple device by the app developers. Participants were then given access to the Feeling Good App, version title “Basic Training Release 2 RCS,” a CBT self-help smartphone app designed to reduce a variety of negative feelings. It was not described as a treatment for any mental disorder or as a substitute for professional treatment, and at sign-up, participants were given resources to pursue professional treatment if desired. The app focused on identifying and modifying negative thoughts and beliefs associated with depression and anxiety and was entirely automated with no human intervention. The app used a 2-phased intervention model, with IBT spanning from 1 to 2 days and then OTMs that required 15 to 30 minutes a day for 4 weeks. The IBT contained modules on cognitive distortions, positive refraining, and relapse prevention and provided psychoeducation in the cognitive behavioral model of mental health disorders. The OTMs taught user strategies to challenge and change distorted thoughts and beliefs.

Participants were randomly assigned to an immediate start (IS) group or a waitlist control (WC) group in a crossover design. This study did not include a true control group, as all participants used the Feeling Good App and were included in the data set. Randomization was conducted using the percentage rollouts randomization feature in a software called LaunchDarkly [30]. The IS group was asked to select a day when they could devote 4 hours to the app for 2 consecutive days to start IBT and were given access to the app. The WC group was also asked to select a day to start IBT after a 4-week waiting period. Each week, participants completed self-report measures. Participants were assessed frequently throughout the beta test, but for this analysis, only four time points were reported: (1) *preintervention time point*, baseline at recruitment from the app waitlist; (2) *start of basic training (SBT)*, at the start of IBT; (3) *end of basic training (EBT)*, completion of IBT; and (4) *postintervention time point*, at the end of ongoing OBT and 4 weeks after SBT.

### Measures

#### Overview

At the preintervention time point, all participants completed a survey that provided information about the app as well as complete demographic information. The preintervention information was gathered using a Google Survey that was securely collected and monitored by the Feeling Good App development team by their report. All other measures were collected electronically through the digital app. When participants were recruited from the waitlist at SBT, they completed a variety of self-report measures, including the Patient Health Questionnaire-9 (PHQ-9), the Generalized Anxiety Disorder-7 (GAD-7), and 7-Dimension Emotion Sliders (7DES). At EBT, participants completed the 7DES. At the postintervention time point, participants completed the PHQ-9, GAD-7, and 7DES. According to the Feeling Good App development team, users interacted with the service via browser at the time they applied to be in the beta test and in the app during the duration of the beta test. During that time, the information the users sent to the Feeling Good App servers was encrypted over HTTPS. The Feeling Good App servers stored information in a Postgres database. The connection between the servers and the database was encrypted. The database application was hosted in a private stack that did not allow connections except from application servers. The database used encryption at rest so that if an attacker was able to obtain the database, they would not be able to access the underlying information. In addition, all identifying information, such as names and email addresses, was encrypted by the application server, so that in the event an attacker was able to access the database they would not be able to match any data to user identities. All data were deidentified before being transferred securely via Box to the authors.

#### Demographic Measures

Demographic information gathered at sign-up included age, gender, income, marital status, education, and race. Clinical information was gathered from all participants, which included current engagement in psychotherapy or psychotropic medication (at sign-up). All demographic data were collected using a Google Survey at sign-up.

#### Safety

During the preintervention screening, any participants who indicated a “1” or higher on item 9 of the PHQ-9 (“Thoughts that you would be better off dead or of hurting yourself in some way”) were excluded from the beta test and given resources for support. The preintervention screening also included 4 questions: “Have you ever made a suicide attempt in the past?” “Have you ever struggled with suicidal plans or urges?” “Have you ever engaged in self-harm behaviors, like burning yourself, cutting yourself, and so forth?” “Do you sometimes have urges or plans to harm others?” Any individuals who responded positively to these questions were excluded and given resources for support. No other safety data collection or monitoring was performed.

#### Qualitative Feedback

**Figure 1:**
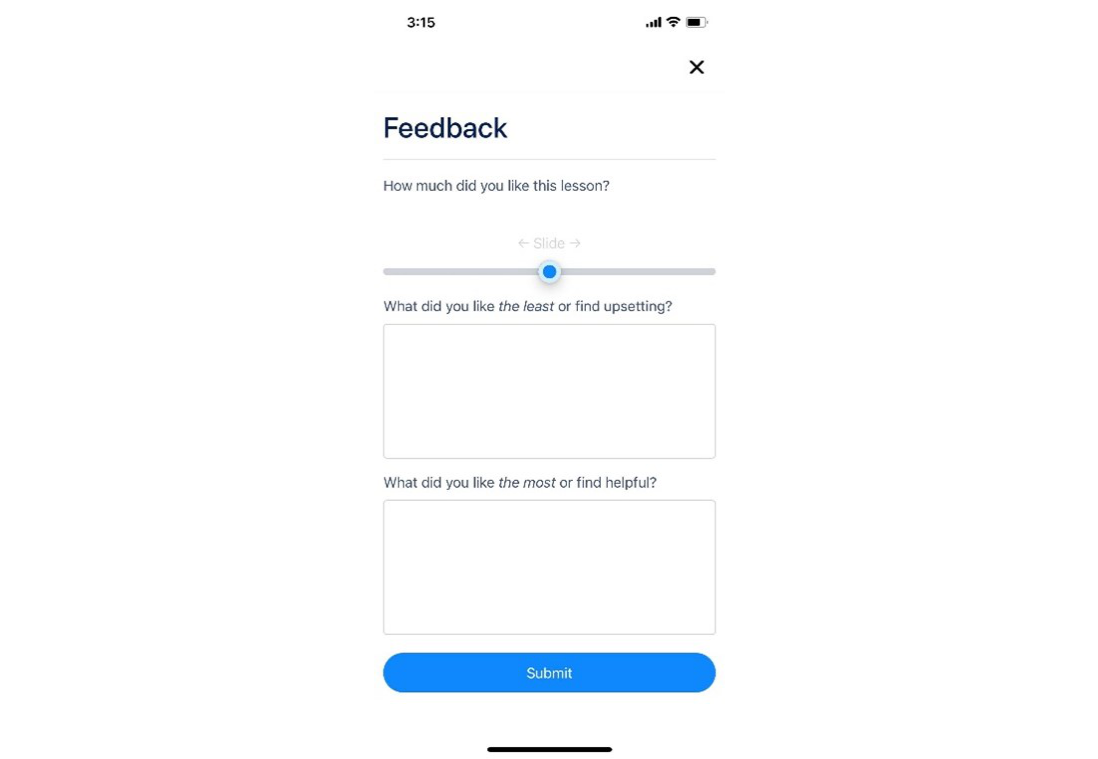
Screenshot of qualitative feedback prompts within the app.

Throughout the beta test, qualitative feedback was collected at the end of every exercise (58 times in total for each individual who completed the full intervention) where participants were asked (1) “What did you like least or find upsetting?” and (2) “What did you like the best?” (Figure 1). Qualitative feedback was not reviewed by the development team until after the conclusion of the study.

#### PHQ-9 Scale

The PHQ-9 [31] is a self-assessment tool that evaluates the degree to which one is experiencing each of the symptoms of depression. This tool uses a Likert scale ranging from 0 to 3 (*not at all* to *nearly every day*) to measure the severity of depression [31]. Summed scores range from 0 to 27, with higher scores indicating greater symptom severity. The PHQ-9 has strong construct validity and reliability as a measure of depression symptom severity [31,32]. Responses were collected via Google Survey during sign-up, and all subsequent responses were collected within the app.

#### GAD-7 Scale

The GAD-7 [33] is a widely used, freely available self-assessment tool that evaluates the severity symptoms of GAD. This tool uses a Likert scale ranging from 0 to 3 (not at all to nearly every day) to measure the severity of anxiety symptoms. Summed scores range from 0 to 21, with higher scores indicating greater symptom severity. The GAD-7 has strong validity [33,34], internal consistency and convergent validity [34,35], and validity as a screening measure for symptom severity [36]. Responses were collected via Google Survey during sign-up, and all subsequent responses were collected within the app.

#### 7DES Scale

**Figure 2:**
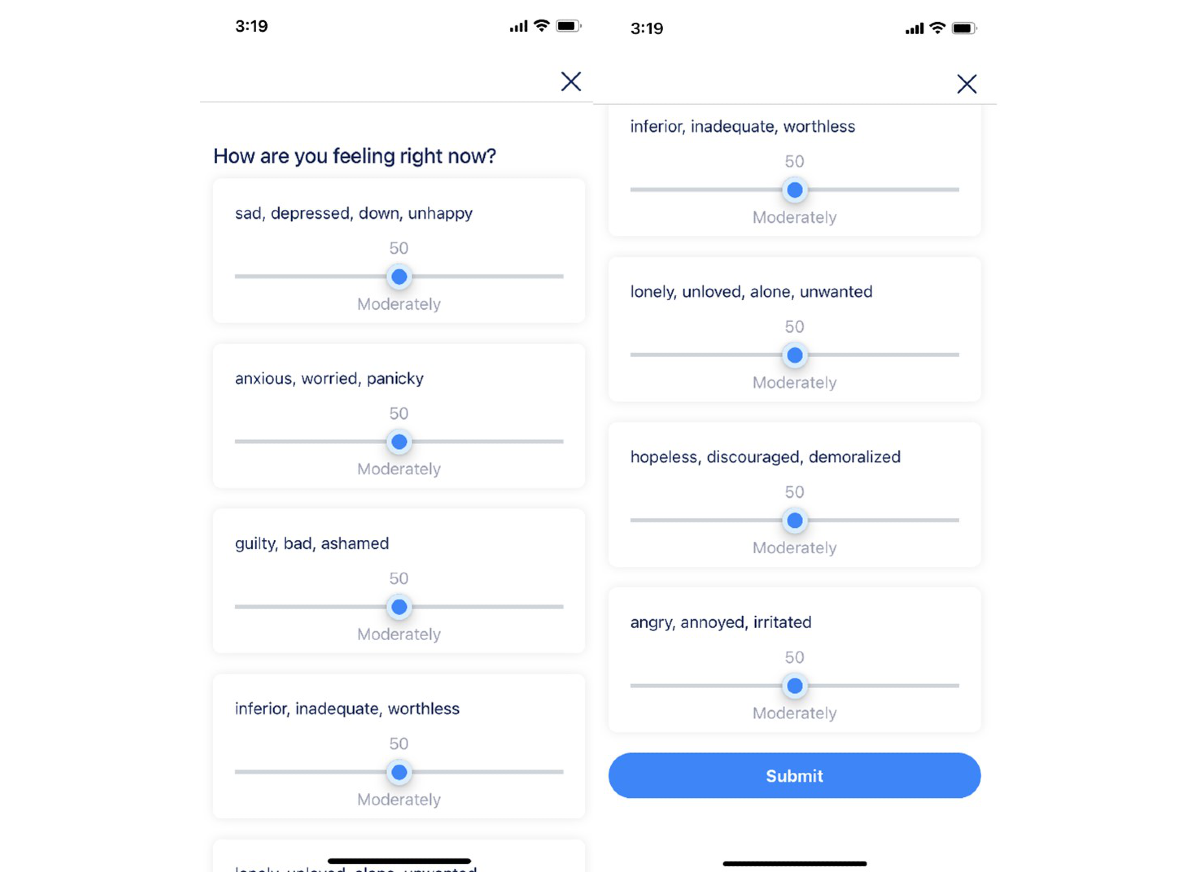
Screenshot of 7-Dimension Emotion Sliders within the app.

The Feeling Good App was designed to produce and track extremely rapid changes in mood. As a result, conventional scales such as the PHQ-9 are not able to measure these types of rapid changes since they ask about the last 2 weeks. Developed for the purpose of the app, the 7DES scale is an ecological momentary assessment [37], which asks users to evaluate on a scale from 0 to 100 the extent to which they are experiencing the indicated negative feelings (Figure 2). These sliders allow for specific report of symptoms, as they change throughout the use of the app. Participants were prompted with the question of “How are you feeling right now?” and asked to move a slider (scale of 0 to 100) to reflect their experience of the following emotions: anger, anxiety, depression, guilt, hopelessness, inferiority, and loneliness. Participants were also asked to complete a single prompt slider rating their current happiness (happiness slider). Participants were prompted with “How happy are you right now?” and provided a 0 to 100 slider to respond (Figure 2). This study examined the data of the full-scaled 7DES-depression (7DES-d) of the 7DES; the anxiety scale (7DES-anxiety) of the 7DES; as well as data from the 5 emotion scales most commonly associated with depression (7DES-composite; depression, guilt, hopelessness, inferiority, and loneliness). Before our analysis, this data set showed that the reliability of this scale at the start of the app was 0.94. The 7DES scale is highly correlated to the PHQ-9 measure (*r*=0.67; *P*<.001). All 7DES responses were collected within the app.

### Data Analysis

All quantitative data were analyzed using SPSS Statistics (version 28.0; IBM Corp). Descriptive statistics were calculated for demographic variables. Feasibility and acceptability were descriptively assessed using the engagement diagram and dropout tables.

Safety was assessed via qualitative and numerical reports provided by Feeling Good App Corporation team developers. Safety was measured quantitatively in 2 ways. First, we directly measured suicidal ideation using item 9 from the PHQ-9, which asks about the frequency of suicidal thoughts over the past 2 weeks. In addition, we measured suicidal ideation indirectly by using the hopelessness slider from the 7DES, which asks about the user’s current feelings of hopelessness. Past research has shown that hopelessness independently predicts suicidal ideation, even when controlling for depression [38,39]. Therefore, we were able to assess safety both directly and indirectly, analyzing changes over time using repeated measures ANOVA.

Efficacy was evaluated using repeated measures ANOVA, which identified changes over time (SBT to postintervention) and by group (IS and WC). The PHQ-9, 7DES-d, and 7DES-composite scores were used to measure depression, while the GAD-7 and 7DES-anxiety scores were used to measure anxiety. Finally, we tested whether baseline characteristics of participant demographics predicted intervention response for both the IS and WC groups by running univariate ANOVA, where the demographic variables were moderators for changes in 7DES scores.

## Results

### Overview

The demographic variables of age, gender, race and ethnicity, highest level of education, income, marital status, current engagement with therapy, and current engagement with psychopharmacology were used. Descriptive statistics were calculated for each group using chi-square test. As shown in Table 1, there were no statistically significant differences between groups for any demographic variable.

**Table 1:** Demographics by group.

Characteristics			Immediate start group (n=141)		Waitlist control group (n=149)		Chi-square (*df*)			*P* value	
Age (years), mean (SD)			45.4 (11.91)		44.05 (12.96)		46.15 (1)			.82	
**Gender, n (%)**								0.01 (2)			.95
	Male	57 (40.4)		61 (40.9)							
	Female	80 (56.7)		87 (58.4)							
	No reply	4 (2.8)		1 (0.7)							
**Race, n (%)**								6.02 (9)			.65
	African American	3 (2.1)		6 (4)							
	Asian (eastern)	5 (3.5)		8 (5.4)							
	Asian (Indian)	3 (2.1)		3 (2)							
	Hispanic	8 (5.7)		6 (4)							
	Mixed race	3 (2.1)		5 (3.3)							
	Native American	1 (0.7)		0 (0)							
	White	105 (74.5)		114 (76.5)							
	Other	8 (5.7)		3 (2)							
	No reply	5 (3.5)		4 (2.7)							
**Highest education level, n (%)**								1.89 (5)			.87
	Grammar or middle school	0 (0)		1 (0.7)							
	High school	2 (1.4)		4 (2.7)							
	Some college or technical training	13 (9.2)		13 (8.7)							
	College degree	26 (18.4)		25 (16.8)							
	Some graduate school	5 (3.5)		7 (4.7)							
	Graduate degree	85 (60.3)		89 (59.7)							
**Income (US $), n (%)**								3.91 (8)			.87
	0-25,000	9 (6.4)		9 (6)							
	25,000-50,000	21 (14.9)		21 (14.1)							
	50,000-75,000	16 (11.3)		21 (14.1)							
	75,000-100,000	24 (17)		25 (16.8)							
	100,000-125,000	14 (9.9)		19 (12.7)							
	125,000-150,000	14 (9.9)		18 (12.1)							
	150,000-175,000	10 (7.1)		7 (4.7)							
	175,000-200,000	6 (4.2)		9 (6)							
	>200,000	27 (19.4)		20 (13.4)							
**Marital status, n (%)**								9.24 (7)			.24
	Divorced	6 (4.2)		10 (6.7)							
	Living together	4 (2.8)		12 (8)							
	Married	77 (54.6)		86 (57.7)							
	Other	2 (1.4)		3 (2)							
	Separated	1 (0.7)		2 (1.3)							
	Single (not partnered)	39 (27.7)		30 (20.1)							
	Single (partnered)	11 (7.8)		5 (3.3)							
	Widowed	1 (0.7)		1 (0.7)							
**Currently in therapy, n (%)**								0.12 (1)			.73
	No	101 (71.6)		104 (70)							
	Yes	40 (28.4)		45 (30)							
**Taking medication, n (%)**								0.66 (1)			.42
	No	104 (73.8)		116 (78)							
	Yes	37 (26.2)		33 (22)							

### Safety

Overall, 207 (40.2%) out of 515 individuals were excluded at screening for suicidality endorsing suicidal urges, suicidal thoughts, and past suicide attempts. For participants of the intervention, on item 9 of the PHQ-9 (“Thoughts that you would be better off dead or of hurting yourself in some way”), there was not a statistically significant change in scores from the SBT to postintervention time point (*t*=0.0, *P*>.99). Although users with suicidal thoughts were excluded from the study at the initial evaluation, at the start of app use, 10 (3.4%) of the 290 users endorsed a 1 (several days over the past 2 weeks) on item 9, while 2 (0.7%) of the 290 users endorsed a 2 (more than half the days). At the end of the intervention, 1 (0.3%) user endorsed a 1 (several days) and 1 (0.3%) user endorsed a 2 (more than half the days).

**Figure 3:**
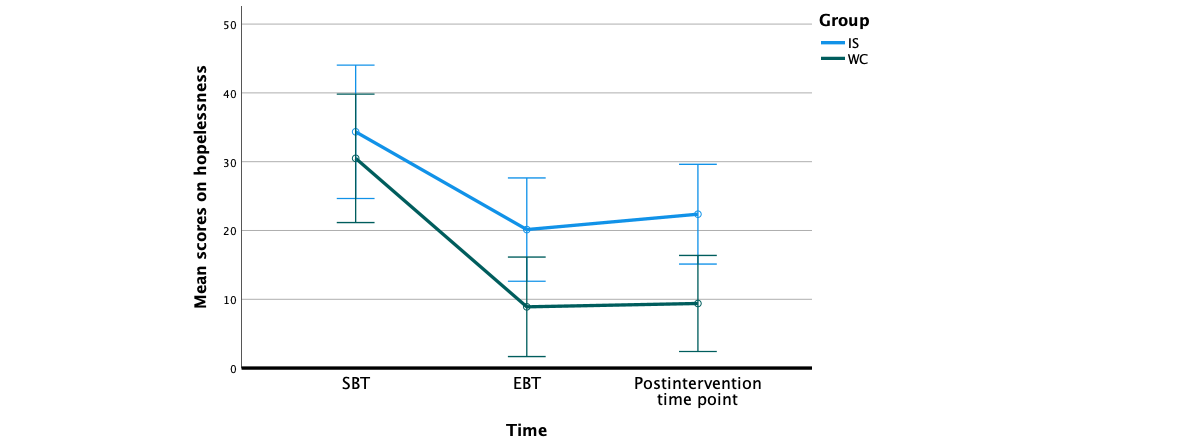
Changes on hopelessness scores from the start of basic training (SBT) to postintervention time point by group. EBT: end of basic training; IS: immediate start; WC: waitlist control.

Throughout the beta test, qualitative feedback was collected at the end of every exercise (58 times in total) where participants were asked “What did you like least or find upsetting?” Qualitative feedback was monitored, and no participants reported endorsed safety concerns throughout the entirety of the beta test. In addition, we measured changes in hopelessness from the 7DES. There was a statistically significant decrease in hopelessness from the SBT to the postintervention time point in both groups (*F*_289_=30.16, *P*<.001), and there was not a statistically significant difference in the hopelessness change between the IS and WC groups (*F*_1_=1.78, *P*=.18; see Table 2 for engagement rates and Figure 3 for changes in hopelessness).

**Table 2:** Engagement rates from preintervention to postintervention time point.

Time point	Total, n	SBT^a^, total (%)	IS^b^, n	IS % of SBT	WC^c^, n	WC % of SBT
Invited to join beta test	4466	N/A^d^	N/A	N/A	N/A	N/A
Started preintervention screening (preintervention time point)	612	N/A	N/A	N/A	N/A	N/A
Completed preintervention screening—enrolled	515	N/A	N/A	N/A	N/A	N/A
Accepted to beta test—randomized	290	N/A	141	N/A	149	N/A
Started intensive basic training (SBT)	230	N/A	113	N/A	117	N/A
Completed intensive basic training (EBT^e^)	166	72.2%	82	72.6%	84	71.8%
Completed ongoing training module (postintervention time point)	80	34.8%	38	33.6%	42	35.9%
Completed follow-up survey	63	27.4%	27	23.9%	36	30.8%

^a^SBT: start of basic training.

^b^IS: immediate start.

^c^WC: waitlist control.

^d^N/A: not applicable.

^e^EBT: end of basic training.

### Feasibility and Acceptability

#### Overview

Invitations to join the study were sent to 4466 individuals who were randomly chosen from beta test waitlist using the percentage rollouts feature on LaunchDarkly. Of those who were invited to join the study, 612 (13.7%) individuals accepted the preintervention screening measures. Overall, 515 individuals completed the preintervention screening process, and 225 were excluded from participation.

**Figure 4:**
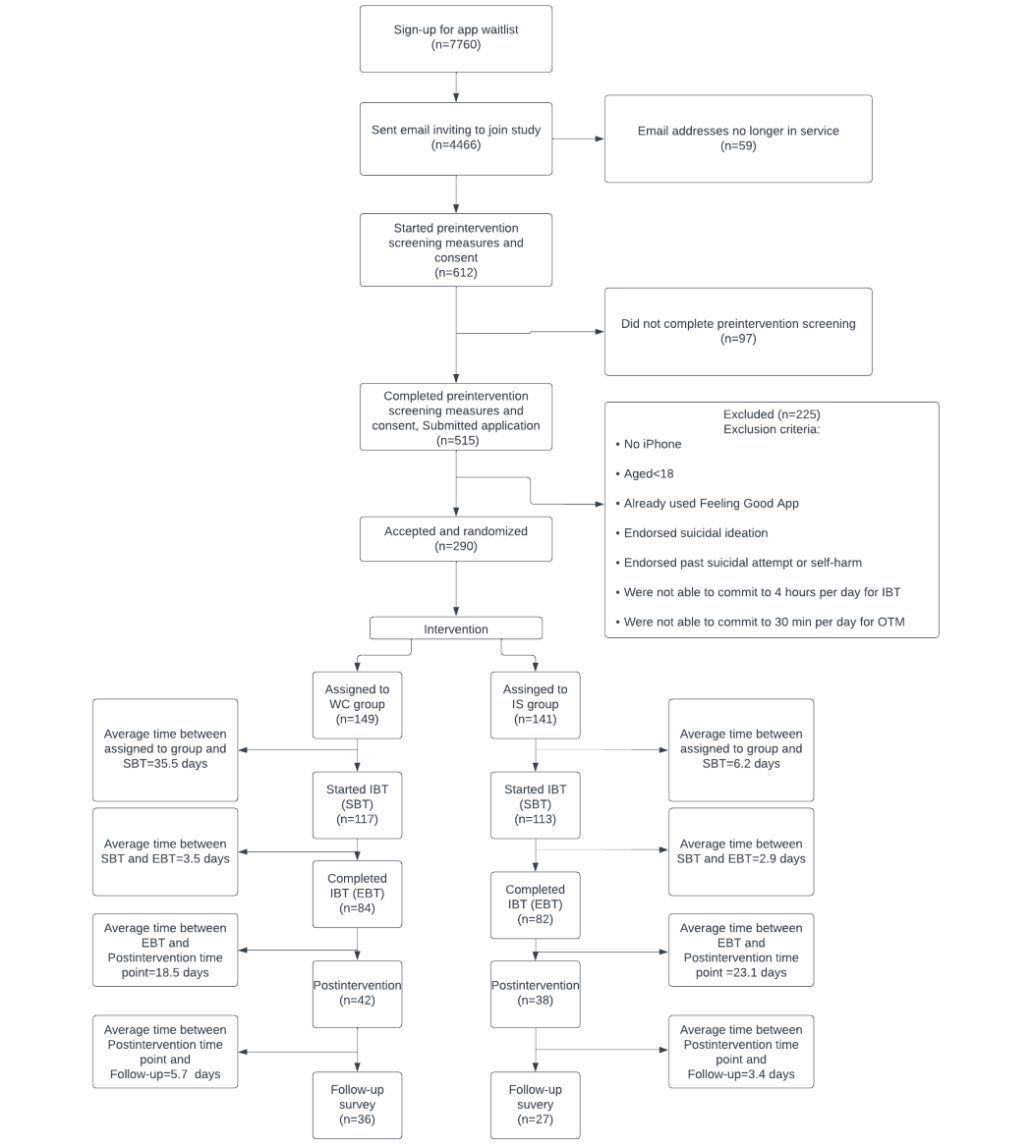
Engagement diagram. IBT: intensive basic training; IS: immediate start; OTM: ongoing training module; WC: waitlist control.

An initial group of 290 individuals were enrolled to participate in the study and completed preintervention screening measures and consent forms. Of them, 141 (48.6%) participants were randomly assigned to the IS group and 149 (51.4%) participants were assigned to the WC group. Furthermore, 113 (80.1%) participants who were assigned to the IS group and 117 (78.5%) participants in the WC group started IBT. Initial enrollment dropout rates for the IS group were 19.9% (28/141) and 21.5% (32/149) for the WC group. Data were not collected about the reason for dropout. Moreover, 27% (38/141) of participants in the IS group completed the postintervention survey, while 28.2% (42/149) of participants in the WC group completed the postintervention survey. A total of 223 participants started IBT, 166 (74.4%) participants completed IBT, and 80 (35.8%) participants completed OTMs. Dropout rates from SBT to EBT were 27.4% (31/113) for the IS group and 28.2% (33/117) for the WC group. From the EBT to postintervention time point, the IS group had a dropout rate of 54% (44/82), while the WC group had a dropout rate of 50% (42/84). Over the 4-week period that users engaged with the app, there was a total dropout rate of 66.4% (75/113) for the IS group and 64.1% (75/117) for the WC group. In total, of the 290 participants who were randomly assigned to groups, 166 (57.2%) users completed IBT, while 80 (27.6%) users completed the 4-week OTMs (see Table 2 for engagement rates and Figure 4 for CONSORT [Consolidated Standards of Reporting Trials] diagram).

#### Qualitative Feedback

Qualitative feedback, gathered at the end of each exercise throughout IBT and OTMs, was reviewed to identify content-based themes. One prominent theme that emerged pertains to users’ expectations and engagement with the app’s structure and pacing. Some users expressed satisfaction with its user-friendly interface, while others raised concerns about its usefulness in addressing intricate psychological challenges. This theme underscores the significance of aligning app design with users’ diverse needs and preferences. Many users expressed appreciation for the app’s educational value, finding tremendous benefit from the lessons, tools, and techniques offered. However, the theme of personalization resonates strongly in users’ comments, highlighting their desire for content tailored to their unique experiences.

Technical glitches and functional concerns represent another notable theme. Users’ reports of technical issues underscore the need for rigorous quality assurance to ensure smooth app functionality. This theme also emphasizes the critical role of seamless user experiences in maintaining engagement and fostering positive change in how they are feeling. Furthermore, users’ reflections on the app’s engagement dynamics revealed its potential to evoke hope and encourage proactive self-care behaviors. Users shared hopeful sentiments about their ability to apply learned techniques from the app into their daily life, and many users looked forward to a commercial release of the app to share with others.

Some users highlighted areas where the app’s pacing and content may not fully align with their preferences, suggesting the need for dynamic content delivery strategies that accommodate varying user needs. One area of concern for many users was the time commitment expected for the completion of the IBT portion of the modules. Results from the data analysis showed that the IBT brings forth some of the most foundational and persistent decreases in symptoms although users report difficulty in committing to the app’s daily requirements of multiple hours.

Upon completing the beta test, many users reflected on the personal growth and positive changes that they experienced throughout both IBT and OTMs. Users expressed a sense of pride and accomplishment and shared how they found the app to be incredibly effective in solidifying their understanding of CBT concepts and skills. Many users reported anticipating using the app again in the future, signaling a desire to continued engagement among users.

### Efficacy

#### PHQ-9 Scale

**Figure 5:**
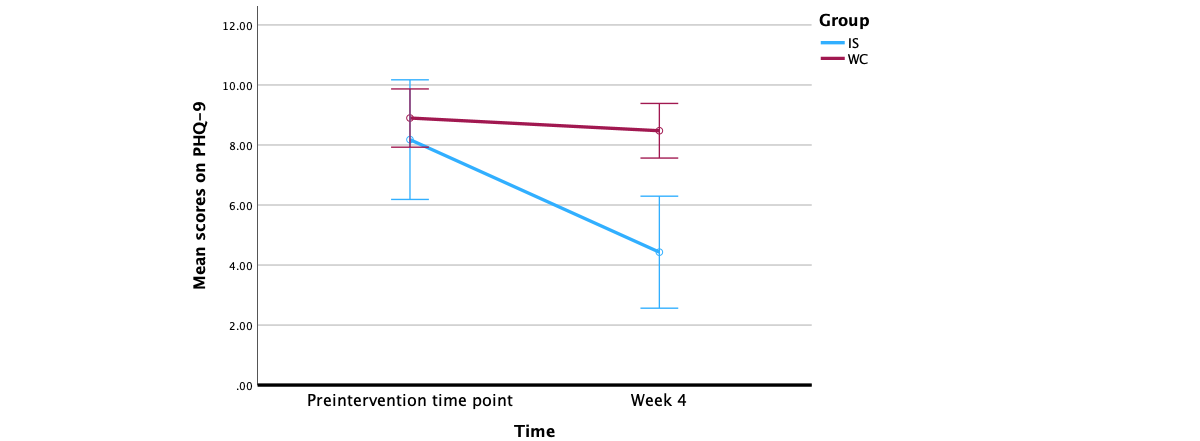
Change in Patient Health Questionnaire-9 (PHQ-9) scores over 4 weeks. IS: immediate start; WC: waitlist control.

We examined the possible effect of the Feeling Good App intervention on depression scores as measured by the PHQ-9, a standardized measure of depression. Using a repeated measures ANOVA, we found that there was a statistically significant within-groups effect of time, where mean depression scores measured by the PHQ-9 decreased from sign-up to 4 weeks later (*F*_197_=31.30, *P*<.001). In addition, there was a between-group effect (*F*_1_=19.88, *P<*.001). As shown in Figure 5, the IS group saw significant changes (*P<*.001) on the PHQ-9 scores over 4 weeks, while the WC group did not (*P*=.20).

**Figure 6:**
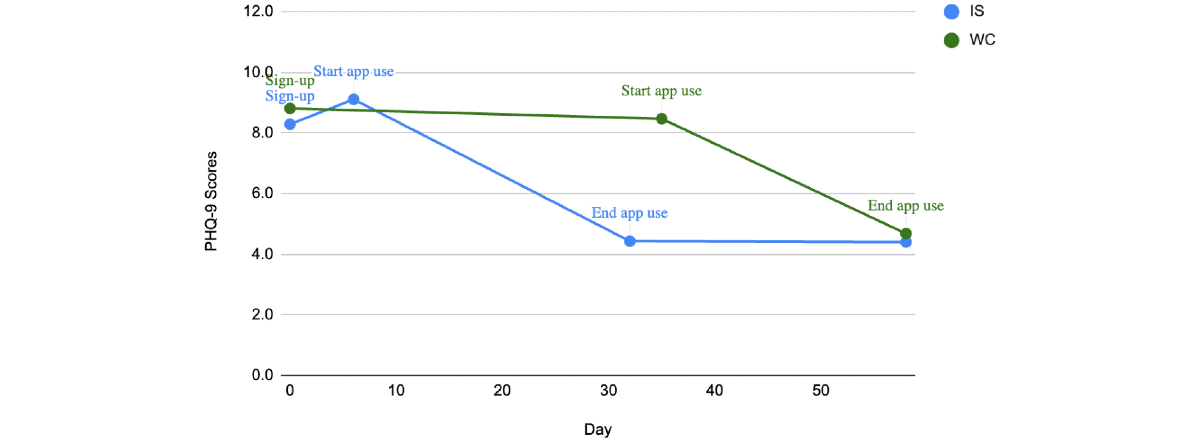
Changes in Patient Health Questionnaire-9 (PHQ-9) from the start of basic training (SBT) to postintervention time point. IS: immediate start; WC: waitlist control.

We then used repeated measures ANOVA to assess changes in PHQ-9 scores from the SBT to the end of the app intervention for both groups. There was a statistically significant difference in PHQ-9 scores from the SBT through the end of the app intervention (*F*_78_=43.680, *P*<.001). Figure 6 shows the change over time for each group, where the WC group saw significant reductions (*P*<.001) in PHQ-9 scores after starting the app intervention.

#### 7DES Scale

In the exploratory analysis, we examined the effect of the Feeling Good App intervention on depression scores as measured by the novel 7DES-d and 7DES-depression measure composite. These slider scales provide information about the severity of current depression symptoms. A repeated measures ANOVA was conducted to measure the changes in depression scores from the SBT to postintervention time point. To account for the participant dropout, analyses for change in time excluded participants who did not complete the measures at both time points. There was a statistically significant change in depression scores throughout the use of the app, where the 4-week Feeling Good App had a statistically significant effect on the 7DES-d (*F*_78_=30.508; *P*<.001) and 7DES-composite (*F*_78_=34.36; *P*<.001).

**Figure 7:**
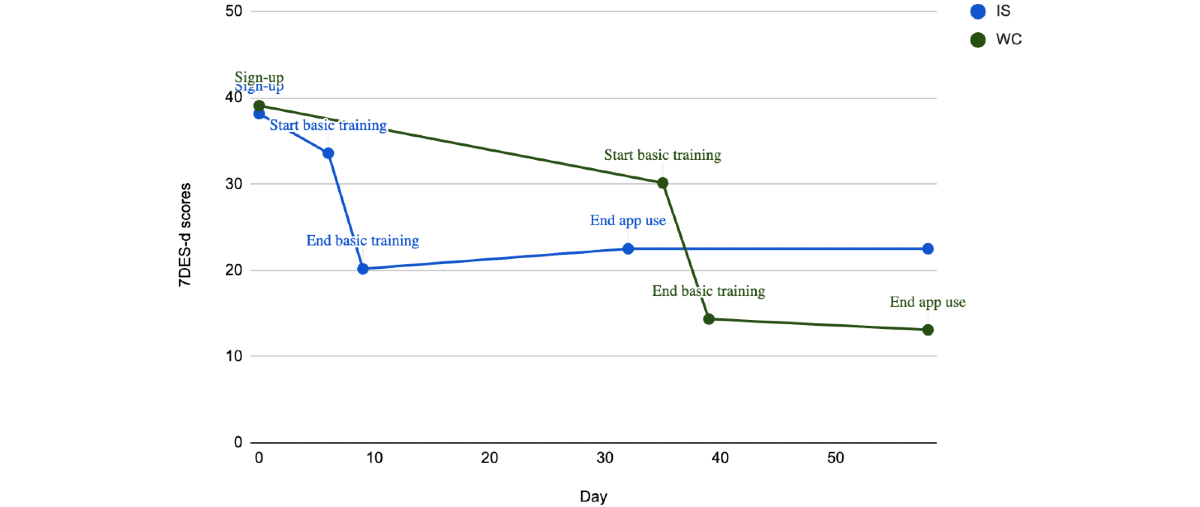
Changes in 7-Dimension Emotion Sliders-depression (7DES-d) scores from preintervention to postintervention time point. IS: immediate start; WC: waitlist control.

**Figure 8:**
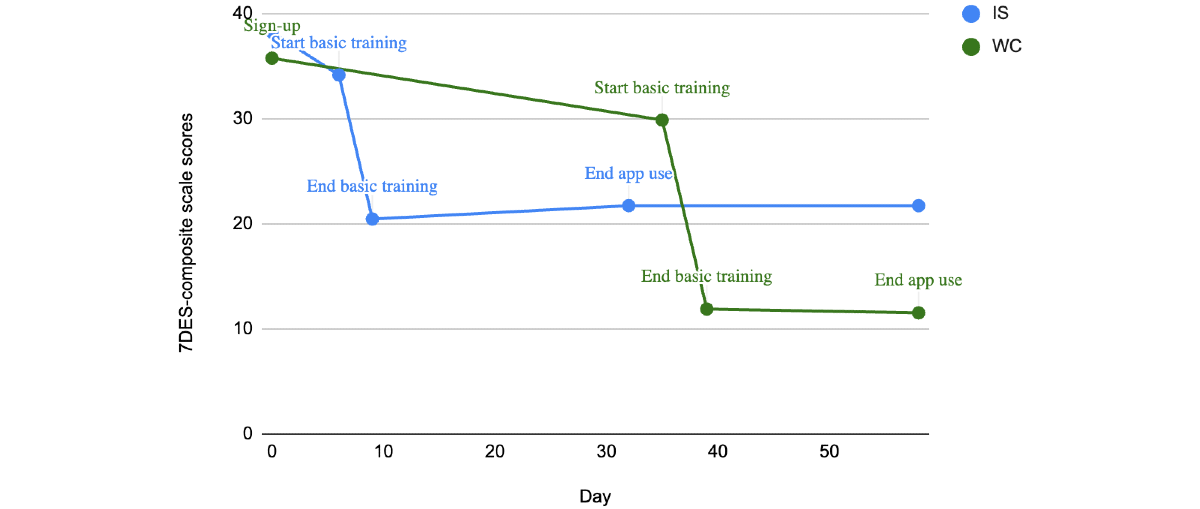
Changes in 7-Dimension Emotion Sliders (7DES)-composite scale scores from preintervention to postintervention time point. IS: immediate start; WC: waitlist control.

Bonferroni adjustment pairwise comparisons were used to measure the change in depression scores between each time point. Table 3 describes the score changes on the 7DES-composite and 7DES-d scores across the intervention period. On the basis of these results, we can conclude that the reductions in depression scores on the 7DES scale occurred during IBT and were maintained throughout the app intervention for both groups. We then measured whether this change over time was different between the groups. In the repeated measures ANOVA, there was not a statistically significant interaction between time and group (*F*_78_=0.81; *P*=.44). Therefore, the group did not impact the changes over time in depression scores, where both the IS and the WC groups saw significant (*P*<.001) decreases in depression scores. Figures 7 and 8 show the change over time by group.

**Figure 9:**
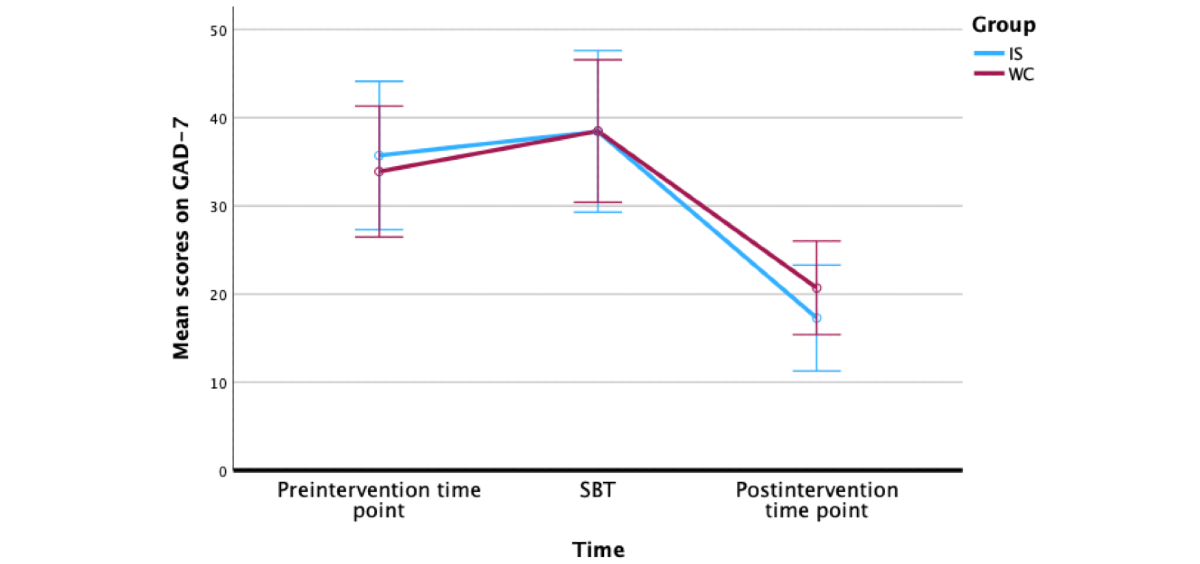
Changes in Generalized Anxiety Disorder-7 (GAD-7) scores from preintervention to postintervention time point. IS: immediate start; SBT: start of basic training; WC: waitlist control.

We also conducted a repeated measures ANOVA to measure the changes in scores on the GAD-7 over time. We found that there was a significant within-participants change over time (*F*_78_=38.99; *P*<.001) and a nonsignificant between-participants effect of group (*F*_1_=0.64; *P*=.53). Using Bonferroni adjustment pairwise comparisons, we found that there was a statistically significant reduction in GAD-7 scores from SBT to postintervention time point for both the IS (mean change 21.14, *P*<.001) and WC (mean change 17.78, *P*<.001) groups (Figure 9).

**Figure 10:**
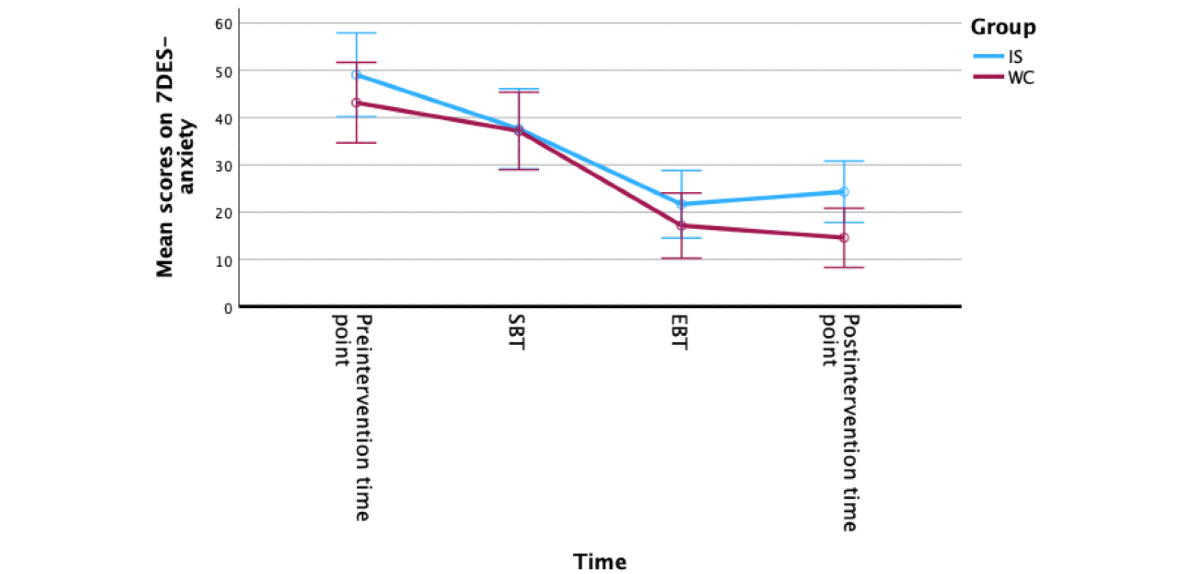
Changes in 7-Dimension Emotion Sliders (7DES)-anxiety item level scores from preintervention to postintervention time point. EBT: end of basic training; IS: immediate start; SBT: start of basic training; WC: waitlist control.

We then measured how the participants’ anxiety changed over time as measured by the 7DES-anxiety. Using a repeated measures ANOVA, we found that there was a significant within-participants change based on time (*F*_78_=50.58; *P*<.001) and that there was not a significant between-participants effect of group on this change (*F*_1_=1.03; *P*=.37). Bonferroni adjustment pairwise comparisons were used to measure the change in 7DES-anxiety scores between each time point. There was a statistically significant difference on the 7DES-anxiety from the SBT to EBT for both IS (mean change 15.92, *P*<.001) and WC (mean change 20.00, *P*<.001) groups (Figure 10).

**Table 3:** Score changes on the 7-Dimension Emotion Sliders (7DES)-composite and 7DES-depression (7DES-d) over time.

Measure				Change across time		*P* value
**7DES-composite**						
	**SBT^a^ to EBT^b^**					
		IS^c^	–13.71		<.001	
		WC^d^	–17.29		<.001	
	**EBT to postintervention time point**					
		IS	1.26		>.99	
		WC	0.27		>.99	
**7DES-d**						
	**SBT to EBT**					
		IS	–14.21		<.001	
		WC	–16.59		<.001	
	**EBT to postintervention time point**					
		IS	0.66		1.0	
		WC	1.24		1.0	

^a^SBT: start of basic training.

^b^EBT: end of basic training.

^c^IS: immediate start.

^d^WC: waitlist control.

### Demographics as Moderators

Finally, we examined the effect of demographic variables on changes in depression scores on the 7DES over time. A univariate ANOVA was used to examine the effect of moderator variables on depression change scores. There were not statistically significant interactions between depression change scores from SBT to EBT and age (*F*_56_=1.11; *P*=.35), gender (*F*_2_=0.02; *P=*.89), race (*F*_9_=0.69; *P*=.63), income (*F*_8_=0.80; *P*=.60), education level (*F*_5_=0.97; *P*=.43), marital status (*F*_7_=1.80; *P=*.13), current therapy engagement (*F*_1_=0.22; *P*=.64), or use of psychopharmacological medications (*F*_1_=0.10; *P*=.76).

## Discussion

### Principal Findings

This study is the first academic and reported proof-of-concept evaluation of the Feeling Good App in terms of safety, feasibility, and initial reports of efficacy. It demonstrates a feasible and novel approach to industry and academic collaboration in the process of developing a digital mental health technology translated from existing evidence-informed treatment. This study may be able to serve as a model for future low-risk, low resource-intensive, minimal conflict-of-interest pathways to conduct initial nonsponsored research with various stakeholders. The results support the initial Feeling Good App prototype as most likely safe for users, having acceptable levels of engagement throughout the length of the intervention and evidence of use correlating with significant decreases in symptoms of depression and anxiety for those with moderate to low levels of clinical symptoms of depression.

The qualitative and quantitative measures of safety demonstrated evidence that this app is generally safe to use when excluding users with current safety concerns before use. Safeguards such as providing resources to individuals who indicated any amount of suicidal ideation on the PHQ-9 and encouraging all users to report concerns of safety allowed for users to proceed with the app without significant safety concerns arising throughout the duration of study. Further consideration of safety for users must be considered as the app moves toward commercial use. These may include protocols in place for allowing those with current or past safety concerns to use and benefit from the app since suicidal behaviors and parasuicidal are unfortunately common in those with mood disorders.

Measures of acceptability demonstrate varying points at which users cease use of the app, indicating that improving the app’s engagement could benefit its widespread usability. App-based mental health tools are often affected by high dropout rates, particularly those that target depression symptoms. Dropout rates for these depression symptom apps tend to fall approximately 47.8%, and apps with smaller samples and more individualized feedback have lower rates on average than larger, generalized samples [34]. Dropout rates in the Feeling Good study can be considered after basic training, when a large percentage of total improvement has already taken place or at the end of the study and follow-up, which would put it above those reported average dropout rates. The dropout rates through the EBT for both groups combined were 28%, which can be attributed to the IBT’s high engagement value and low-time commitment of 1 to 2 days. Over the 4-week period that users engaged with the app, there was a total dropout rate of 66% for the IS group and 64% for the WC group. Further app development may want to focus on improving this dropout, as it is a common problem currently with all forms of treatment for depression and especially for automated digital app– or web-based treatments.

The qualitative data analysis illuminates several salient content-based themes that underscore the multifaceted nature of users’ experiences with the mental health app. These themes encompass users’ expectations, personalization needs, technical functionality, and engagement dynamics. The findings underscore the importance of user-centered design in creating effective digital mental health interventions. The themes identified suggest the potential for integrating advanced personalization techniques, such as artificial intelligence algorithms, to enhance the app’s relevance and resonance with individual users.

Retrospective analysis of this industry-generated existent data set demonstrates a statistically significant (*P*<.001) reduction in depression scores on the PHQ-9 from the beginning of the app to the end of the initial training and to the end of the ongoing modules. However, it is important to note that the cohort was not recruited from a clinical setting, and the mean scores before and after were indicative of mild to moderate depressive symptoms. This study does not fulfill the requirements of a true efficacy study, rather it includes initial statistical impressions interpreted from the data set. The initial training took place over just 2 days, representing the possibility of rapid improvement. Scores from the 2 groups were not significantly (*P*=.41) different at the start of the intervention, so the delayed reduction in depression scores for the WC group aligned with their completion of the app, indicating that changes over time were attributed to app use.

Each of the scales from the 7DES (anger, anxiety, guilt, loneliness, inferiority, and hopelessness) saw significant (*P*<.001) reductions throughout the duration of the initial training. These reductions were maintained through the end of ongoing modules and the users’ time using the app. There were no significant reductions between the end of the initial training and the end of the ongoing modules, suggesting the possibility that the app has the potential to maintain decreased depressive and negative emotions feelings over time.

Reductions as seen through the depression slider 7DES specific to the app were consistent with reductions on the PHQ-9, a popular well-validated assessment tool for depression symptoms. This may support efforts and further exploration of this measure to verify whether it is a clinically and ecologically momentarily valid and reliable measure to be used.

These findings of reduced PHQ-9 and 7DES scores represent potential for this app to help individuals reduce symptoms of depression and associated factors of anger, anxiety, guilt, loneliness, inferiority, and hopelessness. These findings should be further investigated in future studies due to the clinical importance of an app that can help individuals increase their mental well-being.

Individuals from the WC group did not see a significant (*P*=.20) reduction of depressive feelings during the 4-week waiting period. This was surprising, as many times during studies of WCs, there were some improvements seen before beginning an intervention, which may represent expectation, regression to the mean or passage of time, and increased hope for improvement. Research on WC design shows highly variable effects, which depend on the characteristics of sample being studied [40]. One explanation for this lack of waitlist expectation effect in our analysis may be some type of selection bias such as dropout from those individuals who felt their mood improved during the waiting period and chose not to participate. While reasons for dropping out at assignment were not collected, dropout may have been due to remission of symptoms, lack of desire to engage with the program, or technological barriers.

### Limitations

Inherent in any clinical research is the risk of selection bias. The app is currently available by sign-up and is likely reaching audiences familiar with the work of Dr Burns. This bias toward individuals with knowledge related to CBT and psychotherapy may have a moderating effect on the reduction of negative feelings. Future research should take place in naturalistic settings with data from more representative populations perhaps in clinical populations or across cultural and diverse demographic contexts worldwide. The app is not publicly accessible through the Apple App Store and is only available to individuals who are invited to participate in the beta version of the app. With the intention to provide resources at a low cost, these findings should consider the accessibility associated with the app’s current stage of development. In addition, the study population was not a clinical population and showed only moderate severity levels of depression per PHQ-9.

This study occurred over a relatively short period with few follow-up data. One of the primary themes within the qualitative feedback was users indicating a desire to continue to use the skills and lessons provided by the app in the future. Further studies could examine the impact of these modules at a consistent time period beyond the completion of the initial training.

Completion of the Feeling Good App involves active attention and effort, and individuals’ progressions and effort through the completion of the app were not monitored within this analysis. The app can request its users to engage mindfully but cannot account for potential lapses in attention that result in not engaging with the product in its intended context. User retention and engagement are critical to the success of a mobile app–based intervention. While the development team is capable of monitoring engagement, these data were not analyzed in our study. Further research should aim to examine the relationship between time spent in app and modules completed to determine the potential relationship with symptom reduction.

The participants in this study were all given the Feeling Good App and associated assessments, so this study did not include a control group. Future studies that explore the app and its efficacy should compare the users with a control group that is not given access to the app.

Privacy issues were not evaluated, and further assessment of this app using the American Psychiatric Association’s evaluation model and guidelines is encouraged and should include accessibility, privacy, security, clinical foundation, engagement style, and therapeutic goal [41].

Finally, the data analyzed were generated from an industry partner, and there was no way to ensure the data’s accuracy by a third party. Further design and clinical testing will need to have the data collection occur by a third party to improve the level of evidence needed to establish efficacy and effectiveness.

### Conclusions

The following are the summarized conclusions of this study:

- A low-risk, resource-sparing, nonsponsored, and unfunded collaboration between industry and academia is feasible and can occur during the development of an evidence-informed digital mental health app.
- The Feeling Good App in its current form was found acceptable and feasible to deliver to mild to moderately depressed users familiar with TEAM-CBT in a nonclinical setting.
- Safety appears reasonable but may need improvements in assessment, monitoring, and contingency management. Some safeguards are currently in place, while some safety improvements, such as monitoring for suicidal ideation, are needed with commercial use and with clinical populations.
- Acceptability: high dropout scores compared with other apps indicate some improvement in engagement needed.
- Although not designed to test efficacy, this study indicates that those who stay engaged with the Feeling Good App had significant (*P*<.001) reductions in the severity of depression symptoms.
- Evidence of feasibility and efficacy thus far supports further testing and development of this app by a third party using a prospective randomized controlled trial.

## Abbreviations

7DES: 7-Dimension Emotion Sliders
7DES-d: 7-Dimension Emotion Sliders–depression
CBT: cognitive behavioral therapy
CONSORT: Consolidated Standards of Reporting Trials
EBT: end of basic training
GAD-7: Generalized Anxiety Disorder-7
IBT: intensive basic training
IS: immediate start
OTM: ongoing training module
PHQ-9: Patient Health Questionnaire-9
SBT: start of basic training
WC: waitlist control
TEAM-CBT: Testing Empathy Assessment Methods–Cognitive Behavioral Therapy
